# Understanding needs and expectations to start effective communities of practice

**DOI:** 10.1186/s12913-023-10241-z

**Published:** 2023-11-09

**Authors:** Sanne H. Elbrink, Shandell L. Elmer, Richard H. Osborne

**Affiliations:** Centre for Global Health and Equity, School of Health Sciences, Swinburne University of Technology, John Street, Hawthorn, Victoria 3122 Australia

**Keywords:** Communities of practice, Needs assessment, Knowledge translation, Public health, Health literacy, Mental health, Trauma-informed care

## Abstract

**Background:**

Communities of practice (CoPs) are frequently used in health settings to enhance knowledge and support action around public health issues. Yet, most are ineffective and often at risk of not delivering on this promise. To prevent loss of time and resources by organisations, facilitators, and members, this paper argues for a reliable assessment of the needs of people who intend to join and to set realistic expectations to assure effective communities of practice. This research proposes a valid and reliable needs assessment and analysis tool for starting communities of practice, by presenting the results of using such a tool.

**Methods:**

Inception needs assessments were developed, tested and administered to 246 respondents entering five communities of practice that focused on one of three public health issues: health literacy, mental health literacy and trauma-informed care. One community of practice had a global audience, four were based in Australia. Data from the needs assessments were analysed qualitatively and supplemented with descriptive statistics. Results were used to develop an analysis tool to support future communities of practice.

**Results:**

The short-term expectations of respondents included seeking to increase their knowledge and getting to know other members of the community of practice. Long-term expectations shifted towards undertaking action, collaborating and improving health outcomes. While respondents learning expectations included a wide range of topics, they articulated very specific knowledge they expected to share with others. There were high expectations of receiving practical support from the facilitator and a strong preference for meetings with synchronous interaction. Most respondents who planned to join focused initially on the direct and individual benefits and participation they expected from others, whereas they indicated limited intention to actively contribute to the learning needs of other community members. Respondents appeared to need to take time to build self-confidence and trust, and frequently applied a wait-and-see attitude.

**Conclusions:**

The findings of this study suggest that an inception needs assessment allows members to express their needs and expectations, which directly informs the direction and structure of a community of practice, gives voice to members, and supports facilitators in managing expectations.

**Supplementary Information:**

The online version contains supplementary material available at 10.1186/s12913-023-10241-z.

## Background

The United Nations and the World Health Organization are calling for countries, organisations, communities, and governments to take and accelerate action, and work better together to drive innovations in response to public health issues [1, 2]. Communities of practice are a commonly used approach, in and outside health settings, to enhance learning and increase knowledge and are regarded as a promising catalyst towards effective responses to public health issues, including accelerating the implementation of complex programmes [3–5]. The concept of a community of practice has been defined as ‘groups of people who share a concern, a set of problems, or a passion about a topic and who deepen their knowledge and expertise in this area by interacting on an ongoing basis’ [6]. They are typically a low-cost solution, where costs mainly relate to the time commitment of members, technology and meeting expenses [7].

Despite the wide use of communities of practice, there is little empirical consensus on how to best start, structure and run them [8]. Research often includes them as one of many elements in a program (e.g. [9, 10]) or presents them as a solution for a knowledge translation problem, without questioning their utility (e.g. [11]). In addition, communities of practice vary vastly, however, studies often compare the results and processes of different types of communities of practice as if they were the same [8]. Members can interact online, face-to-face or adapt a hybrid combination. Community life cycles can be short-lived or long-lived, and membership can be multidisciplinary or discipline-specific [5]. Studies can, for example, focus on member learning outcomes, practice improvement, common goal achievement, organisational aims, or accelerating interventions [8, 12, 13]. Communities of practice that evolve spontaneously or from the bottom up differ to those that are top-down initiatives. Spontaneous groups often consist of people who passionately start the group and are intrinsically motivated, fostering group ownership. This is usually not naturally present in the more top-down initiated communities of practice, which are often set up to achieve the aim of the initiator, and where members are invited to join [6]. Top-down initiated communities of practice may report positioning problems and a lack of shared aims and interests, with a large share of passive members (lurkers) which can lead to dissatisfied active members [14, 15]. Strong hierarchy and control may also lead to reduced trust and knowledge sharing [13].

Communities of practice rely on people exchanging knowledge and preferring to learn from each other [6]. This suggests that it is important to be aware of people’s knowledge and preparedness to share and learn when initiating a community of practice, however, these elements are frequently ignored by both facilitators and researchers. A small number of studies explored the use of needs assessments or co-designing communities of practice [15–19]. Such needs assessment processes can be time-consuming [16–18] or informal and unstructured [15]. Other needs assessment studies focussed on reasons to be part of a community of practice from an organisational perspective [16], determining the topics to discuss [17] or preferred methods for communication [18]. Another study asked members about their reasons after they already joined the community of practice [19]. Members indicated they did not join for reasons of improving practice or knowledge, but to overcome a sense of isolation through interaction and receiving peer support [19].

To date, community of practice needs assessments tend not to consider the expectations of potential members, including what they might regard as a good return on their investment. This indicates a gap between good practices and research. Meeting people’s needs can increase perceived usefulness by members and can make people feel heard and trusted [20, 21], which in turn, increases the likelihood of useful outcomes. Other potential benefits of a comprehensive needs assessment include management of expectations prior to the start of the community of practice; alignment between topics and members’ needs; and strong group ownership [15]. In this study, we address the identified gap between good practice and research by reporting on the development and application of a process to assess the needs and expectations of potential members at the inception of five communities of practice.

## Methods

This study of developing and applying a needs assessment tool took place at the beginning of a longitudinal study of the setting-up and running of five communities of practice referred to as community of practice A (CoP A) to community of practice E (CoP E, Table 1).

**Table 1:** Overview of the communities of practice participating in this study

Community of Practice	Geographic position	Public health issue
A	Global (18 countries)	Health Literacy and non-communicable diseases (NCDs)
B	State-wide (New South Wales, Australia)	Mental health literacy
C	State-wide (Tasmania, Australia)	Health literacy
D	Nationwide (Australia)	Health literacy
E	State-wide (New South Wales, Australia)	Mental health, trauma-informed care

The five communities of practice in this study had a focus on responding to specific and complex public health issues: health literacy, mental health literacy or trauma-informed care. They all aimed to include members from different organisations (inter-organisational) who could join voluntarily. Four of these communities of practice were based in Australia, of which three were state-based (New South Wales (B&E) and Tasmania (C)) and one was nationwide (D). One community of practice (A) was global (inclusive of 18 countries: Australia, Benin, Brunei, Cameroon, Canada, Denmark, Egypt, England, French Reunion, India, Ireland, Mali, Netherlands, Norway, Portugal, Scotland, Slovakia and Spain) and connected to an initiative of the World Health Organization (WHO). Members of all communities of practice were geographically dispersed. Two communities of practice were connected strongly to existing projects (A & B) and three were derived from existing networks (C, D & E). All agreed to implement the needs assessment and to be observed for a minimum of six meetings. All communities of practice are still active, and evaluation of the longitudinal study is in progress at the time of publication (2023) (Table 2).

**Table 2:** Terms used to describe the roles of different people in this study

• *Initiators* of a community of practice: individual people or organisations taking the initiative to start a community of practice
• *Facilitators* of a community of practice: people who end up running the community of practice and may not be the same individuals as the initiators
• *Members* are participants of a community of practice after joining
• *Respondents* are the people who participate in the community of practice as well as in the research study
• Members often work in or are connected to another organisation for their main role and we refer to their primary organisation as *parent organisation*

Ethics approval for the study was obtained through Swinburne University of Technology Human Research Ethics Committee (reference number 20222875–9323).

### Needs assessment development

The needs assessment for starting communities of practice is intended to serve two purposes: 1. to inform facilitators regarding the needs and expectations of members; and 2. to allow members to voice their particular needs and expectations without feeling group pressure. As there were no known suitable community of practice needs assessments, this study used mixed methods to develop and test an online survey with mostly open questions to obtain an understanding of potentially diverse perspectives [22, 23]. Supplementary file 1 provides an overview of the questions, the rationale for each question, and possible underlying assumptions. This file also describes how the answers were analysed and used by facilitators to co-design each community of practice [23, 24]. After an internal review, the assessment was applied in one community of practice and slightly modified before being applied further.

The needs assessment consists of six parts and is administered before people become a member of the community of practice. The first part started with two questions about expectations about how people think that they, or their parent organisation, might benefit from a community of practice in the short-term and long-term. The second part focused on people’s expectations of the facilitator, of other members and of themselves. The third part included a question about people’s previous experiences in other communities of practice. In the fourth part, we asked people what they wanted to learn and what they were able and willing to share. In the fifth part, we asked practical questions about people’s preferred ways of communication and asked whether there was any type of communication method to avoid. Finally, we provided an option for further comments or suggestions. To help ensure the needs assessment was a low-cost and low-burden tool, we did not collect additional demographic data as these would not be of use to the future facilitators of the community of practice. Table 3 provides an overview of the questions.

**Table 3:** Questions of the needs assessment and data analysis method per question

Question	Data analysis methods
1. In what ways do you think you and/or your organisation or project could benefit from a community of practice? • Short-term benefits (the first three months) • Long-term benefits (one year from now)	Thematic qualitative analysis Descriptive statistics: number of responses per theme
2. For you to get the most out of this community of practice focusing on [insert project name], what, activities, time, commitment and other factors would you like to see in the following: • Other CoP members: • The facilitators of the CoP: • You:	Thematic qualitative analysis Descriptive statistics: number of responses per theme
3. If you have participated in a community of practice before, please describe what worked or did not work for you	Qualitative descriptive analysis Descriptive statistics: positive, negative, number of people with previous experience
4. Is there specific knowledge or experience about the [insert project name] you would like to share with the other members of this community of practice? If yes, please describe: Are there specific things you like to learn more about from other members or facilitators in this community of practice? If yes, please describe:	Qualitative descriptive analysis Descriptive statistics: coded as learn or share
5. What are your preferred ways to interact in this community of practice? Please indicate your preference (1. my most preferred of all, 2. great, happy to do this, 3. doable, 4. not sure, 5. not possible): Online meetings, website, online forum, scheduled chat hours, webinars, email lists (Listserv), closed social media groups or other, name(s)… Is there any software or tool you do not want us to use?	Descriptive statistics: Likert scale
6. Please provide further comments or suggestions to help us co-design the CoP and make it as useful as possible	Qualitative descriptive analysis

### Data collection

Initiators of the communities of practice invited future members to join the community of practice through their regular communication by email. The email included a survey where people could indicate if they wanted to be part of the community of practice and provide informed consent to be part of the research. It was emphasised that people could join the community of practice without participating in the research. People who consented were then asked to fill in the needs assessment. Each initiator sent a reminder. Data were collected in five periods (one for each community of practice) between the end of 2020 and the end of 2021 (Table 4). Initiators received a list of people who signed up to be part of the community of practice. Data were summated by the research team and provided to initiators in an anonymised format. A summary document and presentation of the outcomes of the needs assessment were provided to each community of practice.

**Table 4:** Members, respondents, and response rate per community of practice

Community of Practice	Data collection period	Number of expressions of interest to join the CoP: *member*s	Number of people who consented to be included in the CoP-research: *respondents*	Number of people who filled in at least one question in the needs assessment	Response rate needs assessment in relation to expressions of interest
CoP A	26/08/2021 to 13/09/2021	27	27	19	0.70
CoP B	16/12/2020 to 02/02/2021	50	49	34	0.68
CoP C	30/03/2021 to 28/05/2021	29	29	21	0.72
CoP D	20/06/2021 to 26/08/2021	114	108	84	0.74
CoP E	21/10/2021 to 22/11/2021	129	113	88	0.68
**Total**		**349**	**326**	**246**	**0.70**

### Data analysis and development analysis tool

A sequential mixed method analysis approach was applied, using inductive, thematic analysis and following Braun and Clark’s six-step method [22, 25]. The communities of practice included in this study did not start at the same time, therefore analysis was done individually for each community of practice, so that facilitators could use the results to co-design their community of practice. Each time a data collection period closed, the data were anonymised and uploaded to a qualitative research package (Nvivo12). One researcher (SHE) assigned each answer one or more initial codes. These codes were next merged into potential themes. Each theme was attributed to subthemes and examples of answers from the respondents. The themes and subthemes were then discussed with the other researchers (SLE and RHO) and next with the initiators of each community of practice. Summaries of the findings were presented to the community of practice in their first meeting.

For further analysis, the themes and subthemes of the first four communities of practice (CoPs A, B, C, D) were merged. Next, an analysis tool and codebook were developed (Supplementary file 2), and data from these communities of practice were treated as one comprehensive dataset. The fifth community of practice (CoP E) was used to test and refine the coding process. All the coding was done by one researcher (SHE) and regularly discussed with the other researchers (RHO and SLE). Only higher-order and generic (sub)themes were used for further analysis, as the variance in focus and type of members, meant that some (sub)themes were specific to the topic of the community of practice. Detailed reports were developed for each community of practice separately. Results are presented in this paper in descriptive form and illustrated by quotes linked to a respondent ID number, for example, E62 for CoP E, respondent 62.

The coded data from all the needs assessments were combined since we found no strong thematic differences between the communities of practice. The responses to each question were analysed as follows. The first question about short- and long-term expectations was coded into six themes. Since respondents’ answers were regularly linked to multiple themes, each theme was converted on its own. The same approach was followed for the second question, where respondents were asked about expectations of themselves, others, and the facilitator. The third question involved respondents’ previous experience. This question was coded into positive, negative, or no previous experience. The fourth question included respondents learning needs and the knowledge they wanted to share. Answers were topic specific, so each response was coded as whether respondents wanted to learn something and/or whether they wanted to share something. The last question asked for an indication of communication preferences on a 5-point scale varying from “my most preferred of all” to “not possible for me” on a set of seven different communication methods.

## Results

The initiators received expressions of interest to join the community of practice from 349 prospective members. Of these, 326 prospective members consented to be part of the research project and 246 of them answered at least one question in the needs assessment and 225 respondents answered in full all the questions. Respondents spent less than 15 min on average filling in the needs assessment. Initiators of CoP C and CoP E indicated that a small number of people with lived experience joined the community of practice, which was reflected in some answers, for example, having a history of receiving mental health care. Demographic data were not requested, so the number of participants with lived experience was not obtained. CoP A, B and D consisted solely of professionals. Saturation occurred after analysing the survey responses from CoP D, the fourth community of practice, after which the analysis tool was developed, and subsequently tested with the fifth community of practice (CoP E). There were no major differences observed between the five communities of practice. Table 4 shows an overview of the respondents per community of practice.

### Expected benefits start with knowledge and continue with action

**Fig. 1:**
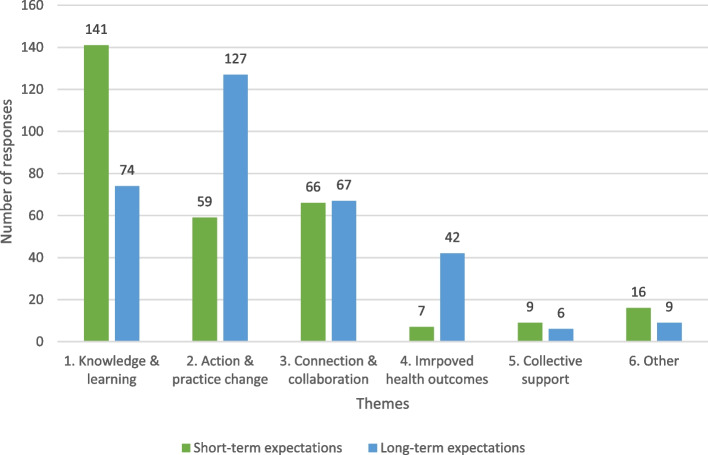
Short- and long-term expectations indicated in needs assessments of prospective members of communities of practice

Respondents indicated ways that they expected to benefit from the community of practice for the first three months and after one year of participation. We observed that prospective members regularly indicated a desire to increase their knowledge for the short term, followed by taking action in the longer term. The analysis identified six themes: 1. increasing their knowledge and learning more about the topic; 2. acting and changing their own or other people’s practice; 3. improving the health outcomes of their patients or clients; 4. connecting and collaborating; 5. getting support; and 6. other expectations, such as being new to the topic and not knowing what to expect. Respondents often indicated expectations in more than one theme. Concerning the short-term benefits (first three months), 235 respondents mentioned 298 different expectations and for the longer-term benefits (over one year) 234 respondents indicated 325 expectations. All identified expectations were treated as having similar weighting. Figure 1 shows the distribution across the various themes. The ‘other’ category includes responses from people who explicitly indicated that they “*don’t know*” or were “*not sure*” what to expect, and occasionally a comment that the respondent was new to the topic and was open to everything. In the next paragraphs, we further illustrate the six themes of expected benefits.

The theme of expectations around knowledge and learning was mentioned more often as a short-term expectation (*N* = 141) versus a long-term expectation (*N* = 74) (Fig. 1). Detailed examination of the data revealed that the subthemes did not greatly differ between the short- and long-term expectations (Table 5). In both the short- and long-term, respondents expected to increase their individual knowledge and understanding about the topic. For the short-term expectations, respondents expected to start improving this knowledge and they used terms such as ‘*improving’*, ‘*expanding’*, ‘*learning’* and ‘*increasing’*. In contrast, for the longer term, respondents expected to have that increased knowledge, which was seen in them using words such as ‘*increased’*, ‘*accepted’* and ‘*have a clear understanding*’. For short-term gains, respondents expected that they would have access to different perspectives or new or very specific knowledge, for example:

*“Hearing different views and understanding what’s important to others, how they feel and think about mental health” (B38 short-term).*

**Table 5:** Overview of themes and subthemes of short and long-term expectations of prospective members

Theme	Short-term expectations (three months from the start)	Long-term expectations (one year from the start)
1. Knowledge & learning	Increasing individual knowledge and understanding Accessing different perspectives and new knowledge Learning about best practices Learning to support others Sharing knowledge and experiences	Having increased individual knowledge and understanding Capacity building Learning about best practices Learning to support others Sharing experiences and resources
2. Action and practice change	Starting to develop (ideas for) action in knowledge development and (organisational) change Building awareness of the public health issues Capacity building	Developing and implementing actions to improve practice and systems Changing culture around the public health issues Creating awareness of the public health issues Supporting the workforce and sustaining motivation
3. Connection and collaboration	Developing networks Connecting with like-minded others Connecting with other (local) organisations	Maintaining and extending networks Connecting with like-minded others and relationships Collaborating and forming partnerships
4. Improved health outcomes	Improving the (delivery of) care	Having improved practice Having improved patient care Having improved health outcomes Getting systemic changes
5. Collective support	Getting project support Getting support in general	Having a feedback and support network Getting project support

Most expectations focused on gaining knowledge and learning from other member’s experiences and the community of practice, for example:

*“Improving knowledge and skills either about Health Literacy either about how a network like this can be used in an effective way for sharing knowledge and experiences” (A15 long-term).*

“*Learning about what is working well in other organisations and being able to take these ideas back to my own organisation with a view to advocating for their implementation.” (E15 long-term).*

Respondents indicated for the short-term that they expected that knowledge sharing would happen, but it was usually not clear if they also expected themselves to share knowledge, for example:

*“Sharing ideas to support project implementation. Support, information sharing and ideas generation during the start-up phase.” (B24 short-term).*

Respondents also indicated for both the short- and the long-term how they planned to use the new or improved knowledge and that they intended to translate that knowledge to others in their parent organisation, for example:

*“For me to have greater understanding so I can incorporate the concepts to entry level training for paramedics” (E82 short-term).*

For the longer-term expectations, respondents connected having new knowledge as conditional to taking steps towards action and making changes that support the response to public health issues, for example:

*“Increased knowledge and confidence to advocate for and create Trauma-Informed services, improve patient outcomes and improve engagement with the service” (E6 long-term).*

#### Action and practice change to respond to public health issues

The second theme described expectations around action and changing practice in response to specific public health issues. This theme was mentioned more often as a long-term expectation (*N* = 127) rather than a short-term expectation (*N* = 59) (Fig. 1).

Detailed analysis of the subthemes revealed a shift from starting to develop actions for the short term to developing and implementing changes for the longer-term (Table 5). For the short-term benefits respondents expected to gain new insights and to start action, indicated in phrases such as: ‘*generate ideas for change’*, ‘*to begin process of planning’* and ‘*develop’.* For the longer-term benefits respondents expected that action has happened as illustrated by words such as ‘*establish’*, ‘*implement’*, ‘*apply’* and ‘*embedded’*.

For the short-term expectations, there was a prospect that the community of practice could support the first steps of action in knowledge development or change in the organisation. The longer-term respondents indicated an expectation of actual improved practice, resources, strategies, and plans, for example:

*“Planting "seeds for change" to introduce health literacy as a concept across our organisation” (C7 short-term).*

*“Establish/develop consumer-targeted resources with appropriate levels of health literacy.” (D34 long-term).*

Respondents also expected that the new knowledge coming from the community of practice would be implemented in practice, policies or capacity building. This was extended by some respondents to a desire to evaluate the actions, for example:

*“Implementation of learnings into workforce development through education and guiding local quality initiatives.” (E77 long-term).*

*‘Increased understanding and support for implementing the MHLI* [Mental Health Literacy Initiative] *in our region. Evaluation of implementation across the various organisations (B35 long-term)’.*

System change was also mentioned as a longer-term benefit of participating in a community of practice, as reflected in these quotes:

*“Increased knowledge in causes and consequences of health literacy. Using this knowledge to guide policy and programs” (D58 long-term).*

*“Direction for specific changes to our policies, practices, resources” (C5, long-term).*

Respondents also desired better awareness about the topic of the public health issue, for example:

*“Better awareness of the role of health literacy in improving health outcomes” (D77 short-term).*

For the longer-term expectations, there was a desire for change in organisational culture regarding public health issues, CoP E respondents in particular commented on this, for example:

*“Organisational change within* [organisation], *not just my team. More standardised approached to TIC* [trauma informed care] *among all teams.” (E19 long-term).*

#### Connection and collaboration with other members

The theme of connection and collaboration with other members was mentioned almost equally as a short-term (*N* = 66) and a long-term (*N* = 67) expectation (Fig. 1). Detailed qualitative analysis of the subthemes revealed differences between the short- and long-term expectations. The respondents were more focused on getting to know others and building a network in the short-term; and collaboration and partnerships in the longer-term (Table 5). For the short term, respondents used words such as “*networking*” or “*network*”. For the longer term, the focus was on extending or sustaining that network or doing something with that network. Some respondents were more specific and described their expectation of networking as being with other like-minded people, for example:

*“Connection to like-minded people interested in health literacy”. (C1 short-term).*

This expectation of networking was also sometimes mentioned for the longer term, where respondents also indicated a desire to do something with the network, for example:

*“Connect with like-minded via a network and bounce/reflect with each other.” (D96 long-term).*

For respondents in the more local communities of practice (CoP B, C and E), the expectation was also to connect with fellow local organisations as exemplified by the quote:

*“Ideas from other PHNs* [Primary Health Networks] *on co-designing with hard-to-reach groups. Connection with other services including LHD* [Local Health District]*.” (B35 short-term).*

For the longer-term expectations, collaboration was mentioned by 34 respondents, compared to 7 respondents who indicated this expectation for the short-term. Some respondents expected collaborations to happen through the community of practice, while others used more speculative phrasing where they indicated exploring collaborations or partnerships, such as:

*“Building relationships across multiple sectors to do more cross-systems/sector active collaboration to* = *tangible results.” (C5 long-term).*

#### Improved health outcomes

In the short term, not many respondents expected benefits regarding improvements in health outcomes of consumers, patients, clients, or carers (*N* = 7). In contrast, for the longer-term, many respondents indicated they expected this (*N* = 42) (Fig. 1). It was specifically suggested that communities of practice could play a useful role, for example:

*“Sharing information within a COP can only help strengthen our work and result in better outcomes for consumers.” (B32 long-term).*

The expectation of improved health was regularly described as improving the healthcare service that could then contribute to improved health outcomes, for example:

*“Embedded practices to deliver improved health literacy to our program participants.” (B8 long-term).*

*“Improved quality of care (care has better outcomes, is safer, more equitable, more patient centred and still affordable).” (A6 long-term).*

Some respondents were more specific and indicated benefits for specific groups, for example:

*“People with severe and persistent mental illness will have their health care needs better met.” (B49 long-term).*

*“Improved health outcomes for pregnant women, people with English as a second language and people with chronic disease” (D66 long-term).*

Others indicated an expectation of improved health outcomes through systematic changes, for example:

*“Health service wide good use of population data to review and improve service delivery and outcomes for community.” (D96 long-term).*

#### Collective support to address public health issues

A small number of respondents indicated expected benefits for themselves or their organisation for short-term (*N* = 9) or long-term (*N* = 6) support from others to respond to the specific public health issues (Fig. 1). For the short term, this support was expected to focus on starting up or implementing projects around the topic, for example:

*“Support/ direction to develop a plan to include consideration of health literacy into service delivery.” (D55 short-term).*

The longer-term expectations took the form of collective support, and answers varied from feedback, project support and having a support system, for example:

*“Continued learnings, adaptations and support with evaluation.” (B27 long-term).*

*“Improve our health literacy programs and support others to improve theirs.” (D23 long-term).*

Table 5 provides an overview of the themes and subthemes, divided between short and long-term expectations.

### High expectations of learning from others

Expectations of others and themselves were explored in a question that asked about people’s expectations of peers, themselves (3.2.1.) and the facilitator (3.2.3.), as well as in a question about people’s learning and sharing preferences (3.2.2.). In general, we observed that people had high expectations of learning from their peers, and (practical) support of the facilitators, while having low expectations about their contribution to the community of practice.

#### Expectations of peers versus expectations of self

**Fig. 2:**
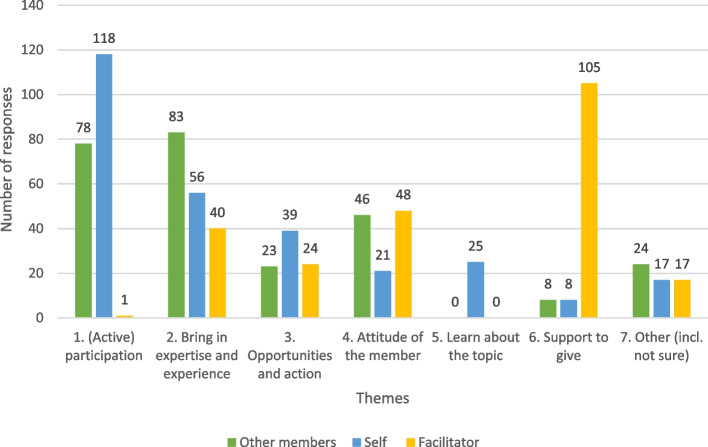
Overview of expectations of other CoP members, themselves and the facilitator

The question about the expectations of others was answered by 195 of the 246 respondents and led to 262 different expectations. Expectations of self were answered by 199 of the 246 respondents, leading to 284 different expectations. All identified expectations were given an equal weight. Respondents indicated expectations about: 1. (active) participation in the community of practice; 2. experiences and expertise that could be brought in; 3. creation of opportunities and action; 4. attitude of the member, 5. Learning; 6. Support, to get or to give; 7. other (e.g., type of members) or not sure what to expect. Figure 2 shows the distributions of responses across the various themes.

Many respondents indicated that they expect others (*N* = 78) and/or themselves to actively participate (*N* = 118) (Fig. 2). When respondents described expectations of themselves, they often used slightly more passive terms like *“attending*” and *“participating*” or *“commitment of time*”. When describing their expectations about others, respondents used more active descriptions like *“active contribution”*, *“regular attendance”* and *“engaged participation”.*

Regarding expectations about the input of expertise and experiences, respondents expected more of others (*N* = 83), compared to themselves (*N* = 58), and yet there was little difference in the way respondents described this theme about others compared to themselves. Respondents expected themselves and others to share knowledge, ideas, experiences, and results. Respondents of CoP A, D and E mentioned this more often compared to people in CoP B and CoP C. In CoP E, respondents with lived experience explicitly indicated their expectation of sharing their lived experiences, for example:

*“As I have research experience and lived experience expertise […] I will give a lot of time, lived experience expertise and be interested in research” (E45).*

There was a difference in how often expectations for (new) opportunities and action were mentioned, where 23 respondents indicated this expectation of others and 39 expected this of themselves. Respondents in CoP B and CoP C indicated more often expectations of themselves around action, while they indicated this less of others. This was opposite to respondents in CoP A, D and E who indicated this less often about themselves and more often about others. From their peers, respondents expected collaborations, involvement in future projects and the creation of opportunities:

*“Research collaborations – specifically in Rural, Remote and Aboriginal communities” (D88).*

*“That all members have trauma-informed approaches in all their work” (C11).*

Respondents’ expectations of themselves were more focused on using the knowledge from the community of practice for their own or the benefit of their parent organisation, for example:

*“To focus on changing practice to be more trauma-informed. To be diligent in interpreting scenarios and feeding back. To be committed to improving TIC [trauma informed care] in my context and feedback what has worked and what has not” (E7).*

A description of attitude was more often mentioned about others (*N* = 46) than about themselves (*N* = 21). Respondents from CoP B and C indicated more often a description of attitude compared to respondents from CoP A, D and E. Respondents described the expected attitude of others and themselves by using descriptions such as *“open”*, *“honest”, “respectful”* and *“willingness to share and contribute”.*

Respondents indicated 25 times that they expected themselves to learn from the community of practice, however, respondents never indicated that they expected others to learn as well. Respondents also expected others to give support (*N* = 8) or to give support themselves (*N* = 8). When respondents indicated a diverse range of ‘other’ expectations (*N* = 13), we observed expectations about the community of practice being a diverse group. Some respondents were not sure what to expect and made that explicit, both in relation to expectations for themselves (*N* = 14) or from others (*N* = 11).

#### General learning needs versus detailed sharing preparedness

Respondents provided information in the needs assessment about topics they wanted to learn more about and topics they could share with the community of practice. Of the 246 respondents, 119 people indicated something they wanted to share, and 184 indicated something they wanted to learn. Among them, 53 respondents indicated that they specifically did not know what they could share, and 15 respondents indicated that they did not know what they wanted to learn. The topics respondents indicated were not coded as general themes, as they tended to be too specific to the different public health issues of each community of practice. Respondents wanted to share topics that usually involved very specific, detailed knowledge of their own experience about responding to a public health issue, and this included respondents with lived experiences, for example:

*“I am involved in the district delivery of trauma informed care to both mental health nursing staff as well as community / allied health staff. As a peer worker I bring a lived experience to enrich the learnings of the sessions. This also helps build empathy for consumers for staff as well as reduce stigma” (E9).*

When respondents indicated they wanted to learn something, it was often phrased in more general terms or the desire to learn from others about what did and did not work:

*“How other members set-up and how organisations started Health Literacy Projects” (A18).* “*Barriers and enablers for implementing projects. What methods work. For those that use co-design what methods work, what is challenging” (B34).*

#### Preference for synchronous communication and practical support by a facilitator

Respondents indicated their expectations of the facilitator, which are summarised in Fig. 2. The question about the expectations of the facilitator was answered by 193 of the 246 respondents and yielded 243 different expectations. There were 105 answers where respondents expected a facilitator to support the community of practice, or, in some cases, support the individual members directly. This support was often indicated as practical support in terms of planning and organising the meetings, facilitating updates or providing communication platforms. Respondents also framed the expected support explicitly in terms of creating opportunities by the facilitator, such as involvement in (research) projects, funding, or collaborations (*N* = 24). Of the 193 respondents, 40 respondents expected the facilitator to have a strong topic expertise or knowledge. The attitude of the facilitator was also regularly described (*N* = 48) as expectations of quality with the more common descriptions being: *“clear communication*”, *“strong facilitation”, “leadership”, “openness”, “guidance”, “knowledge sharing”, “responsive”* and* “supportive”.*

**Fig. 3:**
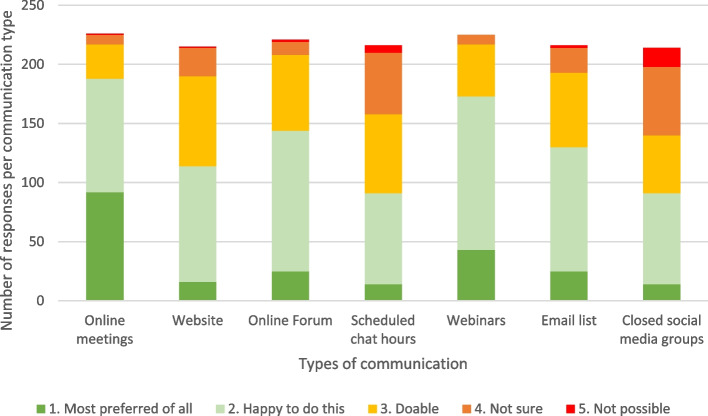
Communication channel preferences

Respondents indicated their preferences for interaction and the use of communication channels. All communities of practice in our study were planned to run online, partly because of their geographically dispersed members and partly because COVID-19 public health restrictions prevented face-to-face meetings, forcing the communities of practice to interact online. While functioning in an online environment, some respondents indicated in the open textbox their preference for an occasional face-to-face meeting. The preferences on seven communication types were indicated by 230 respondents on a scale of 1, “most preferred of all” to 5, “not possible for me” (Fig. 3). Among these, only five respondents indicated their preferences for a few communication types, whereas almost all respondents (*N* = 225) indicated their preference for all seven communication types. Online meetings and webinars were the most preferred options for communication among respondents, followed by online forums, email lists and a website. Synchronous scheduled chat hours and closed social media groups were the least favourable and some respondents indicated that social media should be avoided. Well-known platforms such as Zoom, Microsoft Teams, SharePoint and Basecamp were mentioned as possible platforms to use.

Of the 121 respondents who reported their experiences in previous communities of practice, 101 respondents described what worked well for them in previous communities of practice. 43 described what did not work well. Another 86 respondents of the 246 respondents indicated they did not participate in a community of practice before and three respondents indicated that they participated before (without elaborating on their experience). The other 39 respondents did not answer this question. We did not find any clear relationship between how respondents answered the question about their previous experience to how they answered any of the other questions. When respondents described their previous experience, it was mostly related to the facilitation and organisation of the community of practice, for example:

*“Active, timely, good old use of time. Not so great when meeting too long and only a few are active. Should be relevant” (D23)*.

Respondents also mentioned experience and expectations related to the attitude of the facilitator or the way the meetings were planned and structured in describing their previous experience, for example:

*“Allowing each person to speak and provide input” (B47)*.

*“What worked: good open communication and sharing. What didn't work: People looking for work/consultancies - having another agenda” (C12).*

## Discussion

This study demonstrated a straightforward and inexpensive way to uncover the needs and expectations of prospective members through conducting a needs assessment, which at the same time provided insights into people’s needs and expectations to be used by facilitators for co-designing a community of practice. Diverse individuals expressed their interest in joining a community of practice with a focus on a specific public health issue. Prospective members of five communities of practice reported their needs and expectations. Strong differences in the expected benefits for the short-term versus the long-term were observed. Respondents indicated expectations for the short-term to increase their own knowledge and to build connections, while for the long-term this focus was to ‘do something with the knowledge’ and act, collaborate, change practice and improve care and health outcomes.

Previous research that used needs assessments for communities of practice was scarce and either small and intensive, or retrospective. Our findings were partially in contrast with previous retrospective research that showed that a reason to join was not the desire to increase knowledge or wish to change practice, as people had no interest in changing [19]. Our finding of a focus on gaining knowledge before acting in a community of practice can be explained by the desire to build more confidence in one’s knowledge and capabilities or by a ‘wait-and-see’ approach to see what is in it for them [26, 27]. A focus on connecting and networking before proceeding to collaboration can be explained through the time it takes to build trust between members and uncover common leads to collaborate on [15, 28]. Similar results were found when respondents indicated more often what they wanted to learn, compared to what they wanted to share with the community of practice. The results about the expectations of others, also underpin this, where respondents indicated higher expectations in how others should participate in the community of practice in comparison to themselves. People tend to have a ‘wait-and-see’ approach to new interventions before investing a lot of their time, the need to build confidence and the time it takes to develop trust to open up to others, share and collaborate. The same factors might also explain the high expectations respondents indicate of the facilitation and the ways of working in the community of practice. This result was, however, often in contrast with the wishes of the initiating organisations, who all explicitly indicated in their introduction emails to prospective members and in the first meetings, a preference to transfer the facilitation after a short period to the community of practice themselves. Respondents’ preferences to include synchronous interaction, either online or face-to-face, is consistent with previous research [19, 21] and serves people’s interest in having direct interaction and feedback with other members and facilitation.

Our insights can be used to effectively start a community of practice. We aimed to make the needs assessment a low-burden and low-cost tool, with opportunities for all prospective members to participate and give insights into their needs and expectations. At the same time, the needs assessment supports initiators and facilitators in setting up and running the community of practice more effectively. The needs assessment thus contributes to useful outcomes for both initiators and members in achieving both organisational and individual aims [16, 18]. Utilising a needs assessment makes the community of practice more useful and it avoids wasting time, money, and effort on setting up an intervention that is not achieving its aims [7]. Our needs assessment differs from other assessments in that it is not resource intensive, is more inclusive and focuses on more than the expectations of the parent organisation of members [16–18]. Our aim of developing a useful needs assessment tool for both prospective members and initiators was achieved, as shown by the large share of prospective members that took up the opportunity to respond and were willing to voice their needs and invest an average time of completion of fewer than 15 min. The thematic analysis uncovered recurring themes common to all the communities of practice, which enabled us to develop and refine a helpful tool to assist initiators in the analysis stage of the needs assessment. This analysis tool also reduces time and costs in the analysis stage of the needs assessment for future communities of practice. Our needs assessment is a novel tool as it included the option for all prospective members to voice their needs and included questions about expectations. At the same time, the information gathered guided initiators and facilitators on how to best set up and run the community of practice and it provided insights for expectation management.

The strength of this study is that it provided a first-of-its-kind insight into the needs and expectations of prospective members for the creation of an effective community of practice, supported by the development of a low-burden, low-cost needs assessment and analysis tool. The five communities of practice in our study were all different on several fronts and consisted of a wide variety of people, yet the findings over the five communities of practice were consistent. The robustness of the findings was reinforced through the mixed method study which included both qualitative and quantitative data. The average response rate of 70% is high for health sector professionals, where response rates of less than 50% are common [29]. Initiators estimated that around 95% of the respondents were professionals working in health-related fields. One limitation of our study was that all communities of practice were forced to operate in an online environment due to COVID-19 restrictions or geographical spread. This online restriction might have excluded people with low digital skills, who also did not participate in the needs assessment. One of the communities of practice consists of an international group of professionals. The main language of the community of practice is English, however, some members had limited or no English language skills which may have prevented them from responding to the English-language needs assessment. Another limitation is a possible inter- and intra-coder reliability and confirmation bias that might have occurred by having one researcher undertake all the primary coding and analysis. This was minimised through regular feedback loops within the research team, as well as checking in with members of the communities of practice. The questions about people’s previous experience with other communities of practice did not provide any indications of how to influence people’s attitudes, however, this may occur in the evaluation of the longitudinal study of the five communities of practice. For future research, follow-up with these communities of practice is required to uncover the influence of the needs assessment, as well as to explore how new communities of practice use the needs assessment and analysis tool.

## Conclusion

We conclude that a needs assessment benefits both prospective members and facilitators. Our study provides a much-needed general insight on how to best start communities of practice with a focus on developing individual knowledge and connections by first building trust and then moving towards action on public health issues for the longer term once trust and confidence are established. It is useful for facilitators and members to recognise that high levels of engagement and interaction may not be realistic at the start of a community of practice, and that engagement can grow over time. Initiating organisations should be aware that short-term action on public health issues through a community of practice is unlikely, however, a community of practice has the potential to contribute to medium- or longer-term responses to action towards complex public health issues.

### Supplementary Information

**Additional file 1.** Rationale and questions needs assessment.

**Additional file 2.** Analysis tool and Codebook.

## Data Availability

The datasets used and/or analysed during the current study are available from the corresponding author upon reasonable request.
