# Supplementary material for: Understanding needs and expectations to start effective communities of practice
Source: BMC Health Serv Res. 2023 Nov 9;23:1230. doi: 10.1186/s12913-023-10241-z (PMC10637013; doi:10.1186/s12913-023-10241-z)
Supplement: Supplementary file 1 — Additional file 1. Rationale and questions needs assessment. [file 12913_2023_10241_MOESM1_ESM.docx]

**Supplementary file 1 – Rationale and questions needs assessment**

| **Section A: Your expectations** | | | | |
| --- | --- | --- | --- | --- |
|  | **Question** | **Assumptions** | **Research data** | **Co-design use** |
| A1 | In what ways do you think you and/or your organisation or project could benefit from a community of practice?   - Short-term benefits (the first three months): - Long-term benefits (a year from now): | Members have expectations and can formulate them. | Increase understanding of what people expect before they start. Also, to use to compare later with perceived benefits. | There is often a difference between expectations from members versus the initiator. This helps to make expectations explicit. |
| Rationale: Assessing the expectations can help the facilitator to design the community of practice according to the expectations of the members [1, 2]. It also helps to uncover discrepancies between the expectations of initiators versus members in an early stage and support expectation management. Asking people about their expectations gives people an early sense of community, voice, trust, confidence and a feeling of likely return on investment. This can support active participation in the community of practice that can lead to outcomes of active knowledge exchange and people changing their practice [2, 3]. It also replicates spontaneously evolved communities of practice, where there is often an explicitly shared aim [4]. | | | | |
|  | **Question** | **Assumptions** | **Research data** | **Co-design** |
| A2 | For you to get the most out of this community of practice focusing on [insert project], what, activities, time, commitment and other factors would you like to see in the following:   - Other CoP-members: - The facilitators of the CoP: - You: | Members have expectations and can formulate them. | Increase understanding of what people expect before they start. Also, to use to compare later with perceived benefits. | To make different expectations explicit. Also, to ‘force’ people to make their contribution explicit. It can help the management of expectations. |
| Rationale: This question increases understanding of what members expect of themselves and others. It directly supports facilitators to understand what members expect of them. Strong facilitation, especially at the start, emphasises the importance, improves trust and makes members feel seen. It can also support learning. When learning happens, it is more likely that knowledge exchange and change of practice happen [5-8]. Active participation can support knowledge exchange and change of practice [2, 9] and asking about expectations in participation makes explicit what levels of participation people expect of others versus themselves. Also, dominant individuals or lurkers (passive participants) may reduce the overall active participation of members, so for facilitators, it is useful to know about people’s attitudes beforehand, so they can use that in their facilitation [10-12]. | | | | |

| **Section B: About membership** | | | | |
| --- | --- | --- | --- | --- |
|  | **Question** | **Assumptions** | **Research data** | **Co-design** |
| B1 | If you have participated in a community of practice before, please describe what worked or did not work for you. | Members can remember and are willing to describe this. | Previous experience might impact people’s attitudes and therefore outcomes. | To learn from people’s experiences and to make sure it is positively influencing the CoP. |
| Rationale: Previous experiences might also influence people’s attitudes and expectations when they participate in a community of practice. Experienced members can help with knowledge translation processes, leading to a more likely change of daily practice [13-15]. Experienced members are also more likely to be initial champions, who help to support the community of practice [16-18]. On the other hand, members who are also members of other communities of practice can experience time pressures or conflicting priorities [19]. | | | | |
|  | **Question** | **Assumptions** | **Research data** | **Co-design** |
| B2 | Is there specific knowledge or experience about the [insert specific project] you would like to share with the other members of this community of practice? If yes, please describe: | Members are open and feel comfortable doing this in the new CoP. | If people are willing to share, this might indicate how actively they will contribute to the CoP. It also emphasises reciprocity. | It can give direct input to invite people to share their knowledge, which can be matched with people’s learning needs. |
| B3 | Are there specific things you like to learn more about from other members or facilitators in this community of practice? If yes, please describe: | Members are indeed there to learn and they can make explicit and know what they want to learn. | This gives people a voice and direct influence on the agenda. | It gives direct input about topics to discuss in the Cop. |
| Rationale: These two questions support facilitators directly in uncovering what people’s learning needs are, while it also supports them to know which members might be able to bring in this knowledge. It also gives people the opportunity to influence the agenda [16, 20]. Okafor et al. [21] operationalised this question in their needs assessment by asking participating organisations about the learning needs that benefit the parent organisation. However, it might be more useful to ask the members directly, as there might be discrepancies between what a parent organisation wants, versus what an individual member need. It also misses the opportunity for people to voice their needs. Livergant et al. [22] also included questions about learning topics in their needs assessment about setting up communities of practice for virtual community health navigator groups. Our needs assessment also included a question about what people want to share, to emphasise the importance of reciprocity and mutual interaction exchange direct from the start of the community of practice [4]. | | | | |

| **Section C: Ways of communication** | | | | |
| --- | --- | --- | --- | --- |
|  | **Question** | **Assumptions** | **Research data** | **Co-design** |
| C1 | What are your preferred ways to interact in this community of practice? Please indicate your preference (1. my most preferred of all, 2. great, happy to do this, 3. doable, 4. not sure, 5. not possible):   - Online meetings - Website - Online forum - Scheduled chat hours - Webinars - Email lists (Listserv) - Closed social media groups - Other, name(s)….. | The preferences members indicate correspond with their real preferences. | To uncover the preferences of people about the way they communicate. | To design the community of practice. |
| C2 | Is there any software or tool you do not want us to use? |  |  | To design the community of practice. |
| Rationale: Multiple studies indicated that matching the choice of platform and way of communication with people’s preferences can positively influence outcomes in the community of practice [3, 13, 18, 23]. Misalignment of this may also lead to fewer positive outcomes, even rivalry and competition [24]. Access problems, which can consist of a lack of (digital) skills and technological issues, can lead to less motivation to participate. Livergant et al. [22] also included questions about communication preferences in their needs assessment. Their list is more extensive with questions about people’s usual ways of communication as their communities of practice were established within existing groups, with existing relationships. Our question about communication preferences was asked using a 5-point scale varying from “my most preferred of all” to “not possible for me” for the following options: online meetings, website, online forum, scheduled chat hours, webinars, email lists or closed social media groups. We also included an option for people to add communication methods which were not mentioned. Since all the communities of practice in our research were planned to be online, an option for face-to-face communication was not included. | | | | |

| **Thank you** | | | | |
| --- | --- | --- | --- | --- |
|  | **Question** | **Rationale** | **Research data** | **Co-design** |
|  | Please provide any further comments or suggestions to help us co-design the CoP and make it as useful as possible. | Opportunity to describe other ideas or thoughts they have. | Depending on output. | Depending on output. |

**Reference list**

1. Dijkmans-Hadley B, Bonney A, Barnett SR. Development of an Australian practice-based research network as a community of practice. Australian Journal of Primary Health. 2015;21(4):373-8. doi: 10.1071/PY14099.

2. Bindels J, Cox K, Widdershoven G, Van Schayck CP, Abma TA. Stimulating program implementation via a Community of Practice: A responsive evaluation of care programs for frail older people in the Netherlands. Evaluation and Program Planning. 2014;46:115-21. doi: 10.1016/j.evalprogplan.2014.06.001.

3. Alary Gauvreau C, Le Dorze G, Kairy D, Croteau C. Evaluation of a community of practice for speech-language pathologists in aphasia rehabilitation: A logic analysis. BMC Health Services Research. 2019;19(1). doi: 10.1186/s12913-019-4338-0.

4. Wenger E, McDermott R, Snyder WM. Cultivating communities of practice : a guide to managing knowledge. Boston, Mass.: Boston, Mass.: Harvard Business School Press; 2002.

5. Delbridge R, Wilson A, Palermo C. Measuring the impact of a community of practice in Aboriginal health. Studies in Continuing Education. 2018;40(1):62-75. doi: 10.1080/0158037X.2017.1360268.

6. Mazer B, Kairy D, Guindon A, Girard M, Swaine B, Kehayia E, et al. Rehabilitation living lab in the mall community of practice: Learning together to improve rehabilitation, participation and social inclusion for people living with disabilities. International Journal of Environmental Research and Public Health. 2015;12(4):4439-60. doi: 10.3390/ijerph120404439.

7. Ikioda F, Kendall S. Transformation of health visiting services in England using an Online Community of Practice. Health Policy and Technology. 2016;5(3):298-306. doi: <https://doi.org/10.1016/j.hlpt.2016.02.006>.

8. Abos Mendizabal G, Nuño Solinís R, Zaballa González I. HOBE+, a case study: A virtual community of practice to support innovation in primary care in Basque Public Health Service. BMC Family Practice. 2013;14. doi: 10.1186/1471-2296-14-168.

9. Barwick MA, Peters J, Boydell K. Getting to uptake: Do communities of practice support the implementation of evidence-based practice? Journal of the Canadian Academy of Child and Adolescent Psychiatry. 2009;18(1):16-29.

10. Ford J, Korjonen H, Keswani A, Hughes E. Virtual communities of practice: can they support the prevention agenda in public health? Online J Public Health Inform. 2015;7(2):e222. doi: 10.5210/ojphi.v7i2.6031.

11. Chandler L, Fry A. Can Communities of Practice Make a Meaningful Contribution to Sustainable Service Improvement in Health and Social Care?: Managing Community Care. Journal of Integrated Care. 2009;17(2):41-8. doi: <http://dx.doi.org/10.1108/14769018200900015>.

12. Ortiz-Echevarría L, Mouanga M, Holtz S, Frenchu K. Evaluating Technical Exchange Networks (TENs) at Management Sciences for Health (MSH). Knowledge Management for Development Journal. 2017;13(3):132-48.

13. Ismail A, Kumar N. Empowerment on the Margins: The Online Experiences of Community Health Workers. Chi 2019: Proceedings of the 2019 Chi Conference on Human Factors in Computing Systems. 2019.

14. Langlois EV, Becerril Montekio V, Young T, Song K, Alcalde-Rabanal J, Tran N. Enhancing evidence informed policymaking in complex health systems: lessons from multi-site collaborative approaches. Health Research Policy & Systems. 2016;14:20-. doi: 10.1186/s12961-016-0089-0.

15. Kislov R, Walshe K, Harvey G. Managing boundaries in primary care service improvement: a developmental approach to communities of practice. Implementation Science. 2012;7(1):97-. doi: 10.1186/1748-5908-7-97.

16. Kothari A, Boyko JA, Conklin J, Stolee P, Sibbald SL. Communities of practice for supporting health systems change: a missed opportunity. Health Res Policy Syst. 2015;13:33. doi: 10.1186/s12961-015-0023-x.

17. Shaikh U, Romano P, Paterniti DA. Organizing for Quality Improvement in Health Care: An Example From Childhood Obesity Prevention. Quality Management in Health Care. 2015;24(3):121-8. doi: 10.1097/QMH.0000000000000066.

18. Alcalde-Rabanal JE, Becerril-Montekio VM, Langlois EV. Evaluation of Communities of Practice performance developing implementation research to enhance maternal health decision-making in Mexico and Nicaragua. Implementation Science. 2018;13(1). doi: 10.1186/s13012-018-0735-8.

19. Mabery MJ, Gibbs-Scharf L, Bara D. Communities of practice foster collaboration across public health. Journal of Knowledge Management. 2013;17(2):226-36. doi: 10.1108/13673271311315187.

20. Elsey H, Lathlean J. Using action research to stimulate organisational change within health services: experiences from two community‐based studies. Educational Action Research. 2006;14(2):171-86. doi: 10.1080/09650790600718019.

21. Okafor M, Ede V, Kinuthia R, Satcher D. Explication of a Behavioral Health-Primary Care Integration Learning Collaborative and Its Quality Improvement Implications. Community Mental Health Journal. 2018;54(8):1109-15. doi: 10.1007/s10597-017-0230-8.

22. Livergant RJ, Ludlow NC, McBrien KA. Needs assessment for the creation of a community of practice in a community health navigator cohort. BMC Health Serv Res. 2021;21(1):657. doi: 10.1186/s12913-021-06507-z.

23. Bazyk S, Demirjian L, LaGuardia T, Thompson-Repas K, Conway C, Michaud P. Building Capacity of Occupational Therapy Practitioners to Address the Mental Health Needs of Children and Youth: A Mixed-Methods Study of Knowledge Translation. American Journal of Occupational Therapy. 2015;69(6):1-10. doi: 10.5014/ajot.2015.019182.

24. Rycroft-Malone J, Burton C, Wilkinson J, Harvey G, McCormack B, Baker R, et al. Health Services and Delivery Research. Collective action for knowledge mobilisation: a realist evaluation of the Collaborations for Leadership in Applied Health Research and Care. Southampton (UK): NIHR Journals Library; 2015.
