# Supplementary material for: Understanding needs and expectations to start effective communities of practice
Source: BMC Health Serv Res. 2023 Nov 9;23:1230. doi: 10.1186/s12913-023-10241-z (PMC10637013; doi:10.1186/s12913-023-10241-z)
Supplement: Supplementary file 2 — Additional file 2. Analysis tool and Codebook. [file 12913_2023_10241_MOESM2_ESM.docx]

# Supplementary file 2: Analysis tool and Codebook

This analysis tool can be used to analyse the needs assessment for setting up communities of practice. The analysis tool guides initiators of communities of practice by coding the responses from the needs assessment into generic themes. After this first coding round, the generic themes can be further explored and subdivided into more detailed subthemes. These subthemes are often specific to the context of each community of practice.

## Coding process

A modified version of Braun and Clark’s reflexive thematic analysis [1] can be used for the coding process of the needs assessment.

- Step 1: Read through all the answers to the needs assessment and familiarise yourself with the data.
- Step 2: Import all answers into a software program you would like to use (Microsoft Excel or equivalent is sufficient) and sort them per question (for example, make different tabs for each question in Microsoft Excel).
- Step 3: Analyse each question on its own. Associate each answer to one or more of the main themes in the analysis tool (see [paragraph 1.2.](#_Operationalisation_of_themes)). If an answer fits more than one theme, report them under all matching themes.
- Step 4: For each question, review the answers per the main theme and inductively develop subthemes that resonate with your community of practice. You can use our list of subthemes as a guide, however specific subthemes will likely be identified for your specific community of practice.
- Step 5: Review answers per subtheme, compare subthemes, merge, and refine subthemes, and repeat if necessary. It is useful to do this step with a larger group of stakeholders.
- Step 6: Define and name the subthemes. Go back to steps 3 and 4 to repeat this process for the other main themes, as well as the other questions.
- Step 7: Summarise the findings to share with initiators, facilitators, and members of the community of practice (see [summary tool form](#_Summary_tool)).

If desired, you can add a descriptive quantitative analysis to your findings as well. Our coding form can be found in [paragraph 1.3.](#_Quantitative_coding) of this document.

## Operationalisation of themes and subthemes

Our research has identified the main themes for the questions about short- and long-term expectations, as well as for the questions about expectations of the facilitator, others, and self. The other questions are context specific for each community of practice and need to be inductively analysed. The process above can be followed (exclude step 3).

### Short- and long-term expectations

In the first question, we ask people about the expected benefits for themselves and/or their organisation for the first three months (short-term) after the start of the community of practice and a year (long-term) after the start. People’s answers can be categorised into one of the six main themes. We cannot distinguish between individual expectations or expectations for people’s organisations, as this is often not specified in people’s answers. If people do specify, we can indicate this in the subthemes.

#### Main themes short- and long-term expectations

| **Main theme** | **Description** |
| --- | --- |
| 1. Knowledge and learning | The theme ‘knowledge and learning’ includes all (parts of) answers where people indicate they want to increase, share, exchange their knowledge, get new ideas, learn about experiences, get information, get a better understanding, education, and best practices. Example answers may include:   - *I want to increase my understanding of the topic.* - *I want to learn about the topic.* - *I want to get to know what others are doing.* - *Information and tools about how to implement this.* |
| 2. Action | The theme ‘action’ includes all (parts of) answers where people indicate that they expect that the community of practice leads to some sort of action that goes beyond knowledge sharing and learning. It includes answers that are for example focussed on the process or where people describe what they expect happens with the ‘knowledge and learning’. You can expect answers such as:   - *I expect to change my practice.* - *I want to use it to develop a training for the professionals.* - *I want to teach my colleagues about this topic.* - *I expect that there is more awareness about the topic.* |
| 3. Connection and collaboration | The theme ‘connection and collaboration’ includes all (parts of) answers where people describe how the community of practice connects them with other members of the community of practice and the expected benefits of that. Example answers may include:   - *Networking with ‘like-minded’ others.* - *Get to know who else is working in this space.* - *Future collaborations.* |
| 4. Health outcomes | The theme ‘health outcomes’ includes all (part of) answers where people indicate that they expect that the community of practice leads to better health outcomes for the end-users or better care in general. It is the ‘end-outcome’ or higher-order aim of the community of practice or people’s organisational aims. Example answers may include:   - *I expect to improve care for my patients.* - *I want a better quality of care.* |
| 5. Support | The theme ‘support’ includes all (part of) answers where people indicate they expect to receive (or less likely give) support from the community of practice. This can be support from other members, facilitation, experts, and initiating organisations. This support can be used for their benefit. Example answers may include:   - *I expect support with my project/funding/research.* - *I want help from others with my idea.* - *I am new and expect help from others on how to start.* |
| 6. Other | The theme ‘other’ includes all (part of) answers that cannot be included in the other themes. Most likely these are answers where people indicate they do not have an answer or where the respondent did not answer to the actual question. |

#### Sub-themes short- and long-term expectations

We have described a non-exhaustive list of subthemes we have identified in the answers of the five communities of practice in our research.

| **Theme** | **Short-term expectations**  **(three months from the start)** | **Long-term expectations**  **(one year from the start)** |
| --- | --- | --- |
| Knowledge & learning | Increasing individual knowledge and understanding  Access to different perspectives and new knowledge  Learn about best practices  Learn to support others  Share knowledge and experiences | Increased individual knowledge and understanding  Capacity building  Learn about best practices  Learn to support others  Share experiences and resources |
| Action | Start developing (ideas for) action in knowledge development and (organisational) change  Awareness of the public health issue  Capacity building | Develop and implement interventions and actions to improve practice and systems  Change culture around the public health issues  Create awareness of the public health issue  Support the workforce and sustain motivation |
| Connection & collaboration | Develop networks  Connect with like-minded others  Connect with other (local) organisations | Maintain and extend networks  Connect with like-minded others and relationships  Collaboration and partnerships |
| Health outcomes | Improved (delivery of) care | Improved practice  Improved patient care  Improved health outcomes  Systemic changes |
| Support | Support with (start) projects  Support in general | Feedback and support network  Project support |

### Expectations of facilitator, others, and self

In the second question, we ask people what they expect from the facilitator, their peer members and themselves. Answers can be coded in seven main themes for the facilitator and others and eight main themes for people themselves.

#### Main themes ‘Expectations of facilitator, others, and self’

| **Main theme** | **Description** |
| --- | --- |
| 1. (Active) participation | The theme ‘(active) participation’ includes all (parts of) answers where people indicate where they expect participation, commitment to participation, attendance, contribution to discussions etc. |
| 2. Bring in expertise and experiences | The theme ‘bring in expertise and experience’ includes all (parts of) answers where people indicate that they expect others or themselves to bring in their expertise, knowledge, ideas and experiences, best practices, learnings etc. |
| 3. Opportunities and action | The theme ‘opportunities and action’ includes all (parts of) answers where people indicate an expectation that others or themselves create opportunities through the community of practice and/or undertake action after participation, think about collaboration on (future) projects, find funding etc. |
| 4. Attitude of the member | The theme ‘attitude of the members’ includes all (parts of) answers where people describe something about the attitude or behaviour of people in the community of practice. This is often mentioned when people describe their expectations of the facilitator, sometimes when describing others. For example: inclusive, open, honest, supportive etc. |
| 5. Support to give | The theme ‘support to give’ includes all (parts of) answers where people indicate that they expect others or themselves to give support to other members within or through the community of practice or where people support the community of practice in general. |
| 6. Other | The theme ‘other’ includes all (parts of) answers where people indicate something that does not fit in the other themes. For example, expectations about the type of members involved. |
| 7. Not sure | The theme ‘not sure’ includes all (parts of) answers where people indicate that they do not know or are not sure what to expect. |
| 8. Learn about the topic | The theme ‘learn about the topic’ is so far only found by expectations of people themselves where they indicate the expectation to learn something out of the community of practice. |

We have not indicated set subthemes for this question, as the subthemes are context specific.

## Quantitative coding of the questions

If you choose to do a descriptive quantitative analysis by quantifying your qualitative answers, you can use our coding as a guide. Note: since people can have multiple themes in their answers, you cannot code them as categorical variables.

| **Variable** | **Description** |
| --- | --- |
| Participant ID | Based on CoP_Consent_number: AC1 or ANC2 |
| CoP | A, B, C, D, E |
| WG | Working group member/facilitator/initiator (1=yes, 0=no) |
| NA | Filled at least one question in needs assessment (1=yes, 0=no) |
| STEM1 | Short term expectations main theme 1 = knowledge (1=yes, 0=no) |
| STEM2 | Short term expectations main theme 2 = action (1=yes, 0=no) |
| STEM3 | Short term expectations main theme 3 = connection (1=yes, 0=no) |
| STEM4 | Short term expectations main theme 4 = outcome (1=yes, 0=no) |
| STEM5 | Short term expectations main theme 5 = support (1=yes, 0=no) |
| STEM6 | Short term expectations main theme 6 = other (1=yes, 0=no) |
| LTEM1 | Long term expectations main theme 1 = knowledge (1=yes, 0=no) |
| LTEM2 | Long term expectations main theme 2 = action (1=yes, 0=no |
| LTEM3 | Long term expectations main theme 3 = connection (1=yes, 0=no) |
| LTEM4 | Long term expectations main theme 4 = outcome (1=yes, 0=no) |
| LTEM5 | Long term expectations main theme 5 = support (1=yes, 0=no) |
| LTEM6 | Long term expectations main theme 6 = other (1=yes, 0=no) |
| FAC1 | Facilitator expectations main theme 1 = participation (1=yes, 0=no) |
| FAC2 | Facilitator expectations main theme 2 = expertise (1=yes, 0=no) |
| FAC3 | Facilitator expectations main theme 3 = opportunities (1=yes, 0=no) |
| FAC4 | Facilitator expectations main theme 4 = attitude (1=yes, 0=no) |
| FAC5 | Facilitator expectations main theme 5 = support (1=yes, 0=no) |
| FAC6 | Facilitator expectations main theme 6 = other (1=yes, 0=no) |
| FAC7 | Facilitator expectations main theme 7 = not sure (1=yes, 0=no) |
| OTH1 | Other expectations main theme 1 = participation (1=yes, 0=no) |
| OTH2 | Other expectations main theme 2 = expertise (1=yes, 0=no) |
| OTH3 | Other expectations main theme 3 = opportunities (1=yes, 0=no) |
| OTH4 | Other expectations main theme 4 = attitude (1=yes, 0=no) |
| OTH5 | Other expectations main theme 5 = support (1=yes, 0=no) |
| OTH6 | Other expectations main theme 6 = other (1=yes, 0=no) |
| OTH7 | Other expectations main theme 7 = not sure (1=yes, 0=no) |
| SEL1 | Self-expectations main theme 1 = participation (1=yes, 0=no) |
| SEL2 | Self-expectations main theme 2 = expertise (1=yes, 0=no) |
| SEL3 | Self-expectations main theme 3 = opportunities (1=yes, 0=no) |
| SEL4 | Self-expectations main theme 4 = attitude (1=yes, 0=no) |
| SEL5 | Self-expectations main theme 5 = support (1=yes, 0=no) |
| SEL6 | Self-expectations main theme 6 = other (1=yes, 0=no) |
| SEL7 | Self-expectations main theme 7 = not sure (1=yes, 0=no) |
| SEL8 | Self-expectations main theme 8 = learning (1=yes, 0=no) |
| EXP_POS | Description of positive previous experience (1=yes, 0=no) |
| EXP_NEG | Description of negative precious experience (1=yes, 0=no) |
| EXP_NOT | Indication of no previous experiences (1=yes, 0=no) |
| SHARE_Y | Indication of knowledge and information to share (1=yes, 0=no) |
| SHARE_N | Indication of not wanting/willing/knowing to share (1=yes, 0=no) |
| LEARN_Y | Indication of knowledge and information to learn (1=yes, 0=no) |
| LEARN_N | Indication of not wanting/willing/knowing to learn (1=yes, 0=no) |
| COM1 | Online meetings, 1=most preferred, 2=great, 3=doable, 4=not sure, 5=not possible |
| COM2 | Website, 1=most preferred, 2=great, 3=doable, 4=not sure, 5=not possible |
| COM3 | Online forum, 1=most preferred, 2=great, 3=doable, 4=not sure, 5=not possible |
| COM4 | Chat hours, 1=most preferred, 2=great, 3=doable, 4=not sure, 5=not possible |
| COM5 | Webinars, 1=most preferred, 2=great, 3=doable, 4=not sure, 5=not possible |
| COM6 | Email, 1=most preferred, 2=great, 3=doable, 4=not sure, 5=not possible |
| COM7 | Social media, 1=most preferred, 2=great, 3=doable, 4=not sure, 5=not possible |

## Summary tool

We have developed a summary tool which can be used to summarise the findings and present them to stakeholders and/or the community of practice.

| **1. What are your expectations for how you and/or your organisation or project could benefit from this - short-term benefits (the first three months)** | | |
| --- | --- | --- |
| **Themes** | **Sub-themes + details** | **Example quotes from the survey** |
| **1.1. Knowledge and learning** | [example]  Increasing individual knowledge and understanding   - Learn about mental health literacy - A greater understanding of measurement tools - New evidence-based knowledge - Knowledge about implementation |  |
| **1.2. Action** |  |  |
| **1.3. Connection and collaboration** |  |  |
| **1.4. Health outcomes** |  |  |
| **1.5. Support** |  |  |
| **1.6. Other** |  |  |
| **Quantitative description**  Count the number of answers per main theme (this number can be higher than the number of people who filled in the needs assessment). You can show this in a table. We have presented this in a bar graph, where we combine short- and long-term expectations.  [Example – Figure 2 paper] | | |
| **Summary**  Describe a short summative paragraph of the themes and your findings. | | |

| **2. What are your expectations for how you and/or your organisation or project could benefit from this - long-term benefits (a year from now):** | | |
| --- | --- | --- |
| **Themes** | **Sub-themes + details** | **Example quotes from the survey** |
| **2.1. Knowledge and learning** |  |  |
| **2.2. Action** |  |  |
| **2.3. Connection and collaboration** |  |  |
| **2.4. Health outcomes** |  |  |
| **2.5. Support** |  |  |
| **2.6. Other** |  |  |
| **Quantitative description**  Count the number of answers per main theme (this number can be higher than the number of people who filled in the needs assessment). You can show this in a table. We often present it in a bar graph, where we combine short- and long-term expectations. | | |
| **Summary**  Describe a short summative paragraph of the themes and your findings. | | |

| **3. For you to get the most out of this community of practice focusing on [topic], what activities time, commitment and other factors would you like to see in the following: - The facilitator(s) of the CoP** | | |
| --- | --- | --- |
| **Themes** | **Sub-themes + details** | **Example quotes from the survey** |
| **3.1. (Active) participation** |  |  |
| **3.2. Bring in expertise and experience** |  |  |
| **3.3. Opportunities and action** |  |  |
| **3.4. Attitude of the member** |  |  |
| **3.5. Support to give** |  |  |
| **3.6. Other** |  |  |
| **3.7. Not sure** |  |  |
| **Quantitative description**  Count the number of answers per main theme (this number can be higher than the number of people who filled in the needs assessment). You can show this in a table. We have presented this in a bar graph, where we combine the different expectations.  [Example – figure 3 paper] | | |
| **Summary**  Describe a short summative paragraph of the themes and your findings. | | |

| **4. For you to get the most out of this community of practice focusing on [topic], what activities time, commitment and other factors would you like to see in the following: - Other CoP-members** | | |
| --- | --- | --- |
| **Themes** | **Sub-themes + details** | **Example quotes from the survey** |
| **4.1. (Active) participation** |  |  |
| **4.2. Bring in expertise and experience** |  |  |
| **4.3. Opportunities and action** |  |  |
| **4.4. Attitude of the member** |  |  |
| **4.5. Support to give** |  |  |
| **4.6. Other** |  |  |
| **4.7. Not sure** |  |  |
| **Quantitative description**  Count the number of answers per main theme (this number can be higher than the number of people who filled in the needs assessment). You can show this in a table. We have presented this in a bar graph, where we combine the different expectations. | | |
| **Summary**  Describe a short summative paragraph of the themes and your findings. | | |

| **5. For you to get the most out of this community of practice focusing on [topic], what activities time, commitment and other factors would you like to see in the following: - You/self** | | |
| --- | --- | --- |
| **Themes** | **Sub-themes + details** | **Example quotes from the survey** |
| **5.1. (Active) participation** |  |  |
| **5.2. Bring in expertise and experience** |  |  |
| **5.3. Opportunities and action** |  |  |
| **5.4. Attitude of the member** |  |  |
| **5.5. Support to give** |  |  |
| **5.6. Other** |  |  |
| **5.7. Not sure** |  |  |
| **5.8. Learning about the topic** |  |  |
| **Quantitative description**  Count the number of answers per main theme (this number can be higher than the number of people who filled in the needs assessment). You can show this in a table. We have presented this in a bar graph, where we combine the different expectations. | | |
| **Summary**  Describe a short summative paragraph of the themes and your findings. | | |

| **6. If you have participated in a community of practice before, please describe what worked or did not work for you.** | | |
| --- | --- | --- |
| **What worked well**  **(positive experience)** | **What did not work well**  **(negative experience)** | **Other (not participated, neutral)** |
|  |  |  |
|  |  |  |
|  |  |  |
|  |  |  |
|  |  |  |
|  |  |  |
|  |  |  |
|  |  |  |
| **Quantitative description**  Count the number of answers (this number can be higher than the number of people who filled in the needs assessment, as people can fill in positive and negative experiences). | | |
| **Summary**  Describe a short summative paragraph about your findings; experiences of people, tips for the facilitator or for running the community of practice etc. | | |

| **7. Is there specific knowledge or experience about [topic] you would like to share with the other members of this community of practice? If yes, please describe:** | | |
| --- | --- | --- |
| **Main topics** | **Subtopics + details** | **Example quotes from the survey** |
|  |  |  |
|  |  |  |
|  |  |  |
|  |  |  |
|  |  |  |
|  |  |  |
|  |  |  |
|  |  |  |
| **Quantitative description**  Count the number of answers per main theme (this number can be higher than the number of people who filled in the needs assessment). You can show this in a table. We often present it in a graph. You can also place it next to the topics for people to learn, to see if there is a ‘match’ in topics. Please find an example of one of our communities of practice below: | | |
| **Summary**  Describe a short summative paragraph of the findings. | | |

| **7. Is there specific knowledge or experience about [topic] you would like to learn from other members or the facilitator of this community of practice? If yes, please describe:** | | |
| --- | --- | --- |
| **Main topics** | **Subtopics + details** | **Example quotes from the survey** |
|  |  |  |
|  |  |  |
|  |  |  |
|  |  |  |
|  |  |  |
|  |  |  |
|  |  |  |
|  |  |  |
| **Quantitative description**  Count the number of answers per main theme (this number can be higher than the number of people who filled in the needs assessment). You can show this in a table. We often present it in a graph. You can also place it next to the topics for people to learn, to see if there is a ‘match’ in topics. | | |
| **Summary**  Describe a short summative paragraph of the findings. | | |

| **8. What are your preferred ways to interact in this community of practice?**  **9. Please let us know if you do not want to use or do not have access to specific software or tools.** |
| --- |
| Count the indications per communication type. You can present it for example in a table (see below) or a bar graph (see below).   \|  \| Online meetings \| Website \| Online Forum \| Scheduled chat hours \| Webinars \| Email list \| Closed social media groups \| \| --- \| --- \| --- \| --- \| --- \| --- \| --- \| --- \| \| 1. my most preferred of all \| 31 \| 7 \| 7 \| 4 \| 14 \| 6 \| 3 \| \| 2. great, happy to do this \| 27 \| 34 \| 42 \| 20 \| 43 \| 35 \| 21 \| \| 3. doable \| 9 \| 19 \| 13 \| 20 \| 10 \| 19 \| 10 \| \| 4. not sure \| 1 \| 5 \| 3 \| 17 \| 2 \| 5 \| 23 \| \| 5. not possible for me \| 1 \| 0 \| 0 \| 3 \| 0 \| 1 \| 5 \| \| Total \| 69 \| 65 \| 65 \| 64 \| 69 \| 66 \| 62 \| |
|  |
| **Other input**  Describe if people indicate ‘other’ options. People also regularly indicate communication preferences in the other questions, make sure you include this here as well. |
| **Software not to use**  List all software that people do not prefer to use. |
| **Summary**  Describe a short summative paragraph of the findings. |

| **10. Please provide further comments or suggestions to help us co-design the CoP and make it as useful as possible.** |
| --- |
| List all answers given and where possible transfer to the other questions. |

**Reference**

1. Braun V, Clark V: **Thematic Analysis**. In: *APA handbook of research methods in psychology, Vol 2: Research designs: Quantitative, qualitative, neuropsychological, and biological Edition:.* edn. Edited by Cooper H, Camic PM, Long DL, Panter AT, Rindskopf D, Sher KJ. Washington, DC, US: American Psychological Association; 2012: 57-71.
